# Smartphone Medical Applications for Women's Health: What Is the Evidence-Base and Feedback?

**DOI:** 10.1155/2013/782074

**Published:** 2013-12-18

**Authors:** Emma Derbyshire, Darren Dancey

**Affiliations:** School of Healthcare Science, John Dalton East Building, Oxford Road, Manchester M1 5GD, UK

## Abstract

*Background*. Smartphone medical applications have a major role to play in women's health with their roles being very broad, ranging from improving health behaviours to undertaking personalised tests. *Objective(s)*. Using Medline, Web of Knowledge, and the PRISMA guidelines 15 randomized controlled trials (RCTs) were identified, with mobile interventions being tested on 1603 females, in relation to key aspects of health. Using a similar systematic approach an iPhone database search identified 47 applications (apps) developed to improve women's health. *Findings*. Ten RCTs used text messaging or app interventions to support weight loss, with significant improvements being observed in eight studies. For other aspects of women's health RCTs are needed to determine possible health benefits. iPhone store data analysis identified that a substantial number of women's health apps did not have star ratings or feedback comments (68 and 49 per cent, resp.), raising concerns about their validity. *Conclusion*. Peer-review systems, supporting statements of evidence, or certification standards would be beneficial in maintaining the quality and credibility of future health-focused apps. Patient groups should also ideally be involved in the development and testing of mobile medical apps.

## 1. Introduction

Women appear to be taking the lead when it comes to smartphone technology (ST) phone use, with 56 percent owning a smartphone compared with 51 percent men. This also translates when it comes to using health applications (apps) with around 9 percent women more likely to use these compared with 4 percent men [1]. One American survey studying over two thousand people (*n* = 2020) found that women tend to seek technology that keeps up with their busy lifestyles, with 51 percent owning some form of apple device and 93 percent women keeping their smartphone within arms' length [2].

It is also becoming clear that certain phases of the life cycle for, example, pregnancy, may also affect the level of ST phone usage. For example, one survey of 203 pregnant women found that 94 percent reported that ST had changed their life for better, with 65 percent reporting that they had downloaded pregnancy apps, with an average of three being downloaded during the gestation period [3]. In low- and middle-income countries smartphones also provide an excellent platform to support and improve the quality of healthcare systems for women [4].

There is also growing interest in harnessing smartphone apps to promote behaviour change. These provide a unique opportunity to help users stay healthy, while potentially playing a key role in helping to prevent disease onset [5, 6]. Generally speaking, common modifiable risk factors underlie most major chronic diseases and include tobacco use, unhealthy diets, and physical inactivity [7]. For women especially, rates of overweight and obesity are rising and reaching epidemic proportions, which has broad health implications, including a predisposition towards obesity in the next generation [8].

In terms of other health outcomes, estimated stroke prevalence among women aged 35 to 54 years has tripled over the past two decades, which appears to correlate with increased waist circumferences sizes [9]. Personalised medicine is thought to be one way to target women's obesity prevention, which could consider complex-risk factors such as sex-specific medical conditions and ST could be one approach in terms of helping to deliver this in the future [10].

Other noncommunicable diseases including cardiovascular disease (CVD), diabetes, and cancer have long been leading threats to human health (WHO, 2000) [11]. Looking at the latest statistics, CVD is a leading cause of mortality in women, with over half resulting from coronary artery disease and acute coronary syndromes [12]. Rates of diabetes are estimated to further rise by 2030 with demographic changes such as obesity, sedentary lifestyles, and ageing fuelling the rises in both obesity and diabetes [13]. For female-related cancers, worldwide it has been estimated that more than one million women are diagnosed with breast cancer annually, with more than 410,000 mortalities, indicating that new approaches for preventing and controlling breast cancer, especially in low resource settings in developing countries are still needed [14].

Other health conditions are also generally more prevalent in women. For example, depression prevalence amongst women is almost twice that of men with around 21.3 percent women reporting a lifestyle prevalence of major depressive disorder compared with 12.7 percent of men. Women in their child-bearing years appear to have the greatest risk, with genetic vulnerability, hormonal fluctuations, and psychosocial events such as stress, internalization coping style, and disadvantaged social status thought to affect this [15]. Osteoporosis is also common among postmenopausal women, which increases the risk of fractures, with hip and spine fractures, in particular, being associated with high morbidity and mortality [16].

Given the extent and associated healthcare costs of these conditions preventive health care has the potential to play a key role in reducing the prevalence of these conditions, with approaches such as mobile messaging and app usage offering a convenient and cost-effective way to reinforce desirable behaviours [17]. With the emergence of ST technology now evolving at an alarming pace, it has been anticipated that new software apps will soon be converting smartphones into biomedical devices that are anticipated to play a central role within healthcare systems, potentially turning phones into microscopes, ultrasound machines, or heart-rate monitors [18].

While some RCTs have investigated whether ST use can improve certain aspects of women's health, findings from these have not yet been evaluated systematically. In this paper, we set out to evaluate whether ST use is effective in terms of significantly improving aspects of women's health, including reductions in body weight and improved health awareness for chronic diseases, including diabetes, heart disease, breast cancer, and osteoporosis. The uses of health apps to improve diet quality, support mental, and maternal health are also reviewed. In the second part of this publication, an iPhone database search of health apps developed for women has been undertaken. Data in relation to their cost, star ratings, and user feedback was analysed.

## 2. Materials and Methods

A systematic literature review was conducted, according to the Preferred Reporting Items for Systematic Reviews and Meta-Analyses (PRISMA) guidelines [19] which were used to select the studies and as a guide to the weight placed on these, that is, in this case, only randomized controlled trials were included within the systematic review.

### 2.1. Medline and Web of Knowledge Search

**Figure 1:**
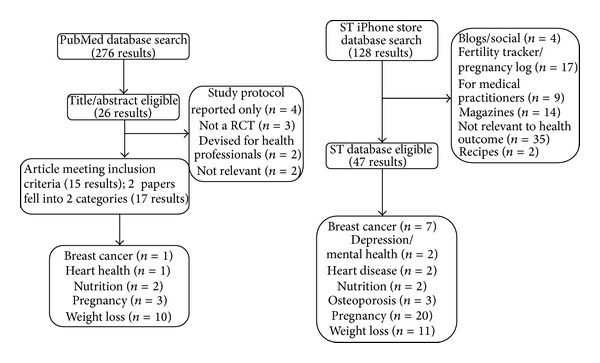
Methodology procedures for Medline, Web of Knowledge, and iPhone store database searches.

The following search strategy was used: Medline and Web of Knowledge databases were searched for English language, peer-reviewed human studies published between August 1983 and 2013 (Figure 1), with the last search run on August 22, 2013. As shown in Table 1 the search was limited to studies looking at “women” and “weight,” “diabetes,” “heart disease,” “osteoporosis or bone health,” “breast cancer,” “nutrition or diet,” “depression or mental health,” and “pregnancy,” combined with “cellular phone,” “mobile phone,” “smartphone or smart phone,” “iPhone,” “android phone,” “blackberry,” “Windows mobile,” or “mobile medical apps”.

For the identification of studies, the search protocol included the following stages: screening of identified papers; examination of the full text of potentially relevant studies; and application of the inclusion criteria to select the included studies. All reports were assessed by ED for suitability. For the study to be included in the main systematic review, the following characteristics were screened and inclusion criteria were applied: (a) studies should use mobile devices to improve aspects of women's health, (b) the intervention group should be compared with a controlled group, (c) the publication must be a randomized trial, (d) the study was not a combined multi-intervention; and (e) access to the full text paper was available, either through Medline, Web of Knowledge or by contacting the author(s).

Papers were excluded if (1) they were a study protocol, (2) applications were for health professionals, (3) applications were appointment reminder systems, (4) the study did not include any measures of women's health or (5) intervention used computer-tracking systems rather than phone devices. The reference lists of scientific papers and reports were also hand searched by ED to identify relevant papers.

### 2.2. iPhone Store Search

The iPhone app store contains around 300,000 apps and it is growing [34]. A mobile iPhone database (application store) was searched with the same combination of terms used in the Medline search: “breast cancer,” “depression/mental health,” “diabetes management,” “heart disease,” “nutrition,” “osteoporosis/bone health,” “pregnancy,” and “weight loss,” combined with “women's health.”

The search was limited to apps specifically developed for women's health, with the following being excluded: (1) apps for health magazines, (2) dictionary/medical term apps, (3) blogging/social apps, (4) apps developed for medical practitioners, (5) apps that did not relate to the specified health outcomes, or (6) apps that were games. In the case of pregnancy apps, those that were period/fertility trackers or pregnancy calendars were also excluded, as these did not relate directly to health.

All included apps had a central function that involved improving health/behaviour or preventing disease development. Once retrieved the ST apps were searched manually by ED for relevance. The last search was conducted on the 22 August 2013. Once relevant ST apps had been identified information in relation to their cost, star ratings and comments were collated. This data was extracted from the reviews section of each app and logged into an excel database for analysis.

## 3. Results 

### 3.1. Medline Results

As shown in Figure 1, from our initial Medline search 276 articles were identified. A title and abstract review was undertaken, from which 26 articles were selected for detailed review, with 15 meeting the eligibility criteria (Table 2). Ten studies were found to investigate the use of ST health apps in relation to weight loss, while six studies focused on other aspects of women's health. No RCTs were found to study the effects of mobile messaging or app use in relation to markers of depression/mental health in women, diabetes, or bone health/osteoporosis.

From the 16 RCTs identified, ten studies have focused on the use of mobile short messaging services SMS/apps to assist with weight loss. Of these, two studies investigated the effects of SMS messages, received over a period of 12 months [30, 32]. One of the largest studies carried out by Shapiro et al., [30] recruited and randomized 111 overweight/obese females to receive either mobile text messages containing facts, tips, and motivational weight loss information or monthly newsletters. Results showed that adherence rates were good (60–69%), with strong compliers loosing significantly more weight at 6 (*P* = 0.39) and 12 months (*P* = 0.23) compared with those who were less adherent/did not follow the messages. A similar study by Haapala et al. randomized 96 overweight females to receive staggered instructions in relation to reducing food intake, with daily weight reporting and feedback over 12 months, or a control group (no intervention). After 12 months the experimental group had lost significantly more weight (4.5 kg) compared with the control group (1.1 kg) (*P* = 0.006) [32].

A further four studies considered the effects of ST use as part of weight loss interventions. Brindal et al. [26] developed an app to support users taking part in a meal replacement programme (MRP). Fifty-eight overweight/obese women were randomized to receive either the MRP support app or a static app (control) for 8 weeks. No statistically significant differences were observed in relation to levels of weight loss, although women in the intervention group reported a greater increase in mood (*P* = 0.012) compared with the control. It is possible that 8 weeks were not long enough for effects to be observed in this study. A longer RCT by Carter et al., [27] conducted over 6 months, randomized 42 females to three groups: (1) app group, (2) website use, or (3) paper diet/physical activities diaries. The app group was able to self-monitor the diet and physical activity levels, with weekly feedback being provided. Findings showed that body mass index reductions after 6 months were the highest amongst the app users (−1.6 kg/m^2^), followed by the diary group (−1.0 kg/m^2^) and the website group (−0.5 kg/m^2^), indicating that this app was acceptable and feasible.

Patrick et al. and Norman et al. also found that weight management texts sent 2–5 times per day, over 4 months led to significant reductions in body weight [23, 33] while other work has shown that self-monitoring of body weight, that is, texting weight regularly to practitioners, or logging body weight measurements into mobile devices can lead to, also led, to significant improvements in body weight/waist circumference measurements [21, 28].

Finally, two RCTs have investigated the effects of ST apps in combination with social media application such as Facebook or Twitter. For example, one study found that while the number of podcasts downloaded was significantly and moderately correlated with weight loss in both the Podcast (*r* = −0.53, *P* < 0.001) and Podcast + Mobile (*r* = −0.46, *P* = 0.001) groups, prompting and mobile communication via Twitter, and monitoring the app without feedback did not appear to enhance weight loss, possibly because the addition of Twitter and mobile monitoring became a distraction [31]. Napolitano et al. randomized 45 female overweight or obese college students to (1) receive weight loss advice either via Facebook, (2) through Facebook, text messaging, and personalized feedback or (3) no advice (control). While it is difficult to separate out the effects of the text messaging and personalized advice, this intervention group was found to loose significantly more weight (2.4 kg) over 8 weeks compared with the other groups [29].

Three studies have investigated the use of mobile text messaging in relation to changing health behaviors in pregnancy but RCTs using apps remain to be understudied. Naughton et al. found that tailor text messaging was more successful in terms of helping women quit smoking during pregnancy, when compared with traditional leaflets containing information [24]. Evans et al. as part of the Text4baby study found that there was an improvement towards alcohol consumption compared with baseline (*P* = 0.029), although further findings are yet to be published from this work [35]. Finally, Jareethum et al. randomized women to receive text messages over 28 weeks, offering general support to women during the prenatal period for example, on how to prepare for having their baby and introducing it to the home environment. This intervention was successful in that confidence levels were significantly higher (*P* = 0.001) and anxiety levels significantly lower (*P* = 0.002) among women receiving the intervention messages [25].

Two studies have investigated the use of mobile SMS messaging in relation to other aspects of women's health. One study randomized 385 women to receive either a general SMS invite to do a mammogram or an invite, combined with additional SMS information about the benefits of mammogram screening. The number of women undertaking a mammogram after the intervention was similar for both groups (31 and 32%). This is still considered to be quite low, which could be attributed to the fact that 26.4% of cell phone numbers were invalid [20]. Another study carried out in Iran where median urinary iodine concentrations have been found to decline (an indicator of iodine deficiency), randomized 205 females to an intervention group receiving daily text messages via their mobile phone for 6 weeks about iodine deficiency and the use of iodised salt or a control group. Levels of knowledge about the topic of iodine deficiency were significantly improved by the end of the study (*P* = 0.004), although this was not reflected by a change in urinary iodine levels [22]. It is possible that the dietary adjustments made were not sufficient to generate a measurable, physiological change. Two studies investigating the effects of mobile messaging on markers of body weight and also identified important secondary findings. Park and Kim found that systolic and diastolic blood pressure and total cholesterol declined in the weight loss group receiving text messages [21] while Norman et al. found that fruit and vegetable intakes significantly improved [23].

### 3.2. iPhone Database Results

**Figure 2:**
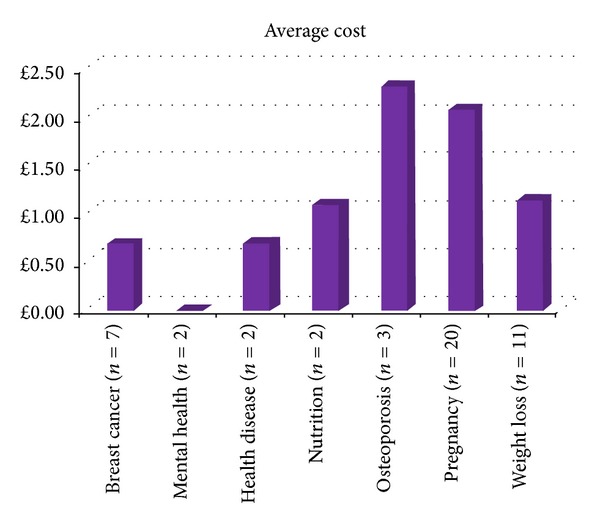
Average cost of women's health apps from the iPhone store. Note: mental health apps were both free.

**Figure 3:**
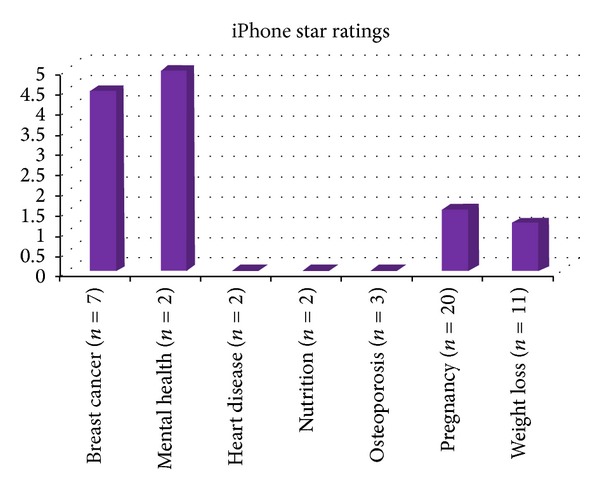
Average star ratings. Note: star ratings were not recorded for the heart disease, nutrition, or osteoporosis apps.

From the iPhone database search, using similar key terms, 128 related health apps were retrieved. However, after evaluating these, only 47 met the specified inclusion criteria, from which data was extracted and analyzed, as shown in Figures 2 and 3.

Just under half (46.8 percent) of the apps meeting the inclusion criteria were free. Of the 53.2% that were paid for, their average price was *£*1.44, SD 1.17. Fifty-six percent of the apps were priced at 69 pence, with the most expensive costing *£*4.50. In terms of star ratings and feedback comments, a large proportion of the apps (68 and 49 percent, resp.) did not have these reported. For those that had been star rated, the average score, out of a possible five was 2.5, SD 2.9. Figures 1 and 2 show the cost and star ratings of apps according to the specified health categories. Clearly, larger and more complete datasets are needed but preliminary analysis indicates that pregnancy apps are generally priced higher, yet appear to have lower star ratings.

For apps where comments were present, an anonymized table of commentaries is included in Table 3. Most of these indicate that users are in favor of apps that are of easy to use, contain new information, and are motivational. Areas in need of improvement appear to be the quality of graphics, speed of downloads, for example, graphs, compatibility with other devices, ability to transfer data on to new versions, and certification/affiliation with credible organisations.

## 4. Discussion

Overall, smartphone technology appears to hold great promise in the future in terms of helping to deliver health behaviour changes [36]. However, the present paper has identified some key areas for consideration. Firstly, in terms of published evidence, while their appears to be several studies investigating the effects of mobile messaging in relation to aspects of women's health, comparatively few studies have tested the efficacy of mobile medical apps.

Given the rise in the number of medical mobile applications there is a clear need to support these with a strong, scientific evidence-base, to ensure they deliver the desired benefits; otherwise, once in the public domain this could impact on user safety. In particular, one review of diabetes apps, downloaded through the iPhone store found that most of these lacked evidence in relation to clinical efficacy, concluding that they would not integrate well within the health care delivery system [37]. Equally, while these hold great potential in terms of improving clinical practice, possible dangers associated with their use have yet to be identified. For example, breaches of patient confidentiality, conflicts of interest, and apps with unclear or inaccurate clinical decisions/outputs could all have negative implications for patient care [38]. Peer-reviewing of medical applications, along with provisional testing by relevant patient groups, could be one way forward [39]. However, until further reaching and testing are undertaken, while the use of medical apps may be appealing, their clinical efficacy is less clear [40].

We have now also reached the era of personalised medicine and nutrition [41]. Given this, it is concerning to see that few apps have been developed with a view to accounting adequately for lifestyle and genetic factors, for example, family history, or tailored specifically to aspects of women's (and men's) health. Of the applications developed in relation to improving aspects of women's health, pregnancy and weight loss appeared to have the strongest presence within the iPhone app store. While these appear to be supported with some level of scientific evidence in the form of RCTs, given the number of apps for pregnancy available through the app store, this is something that should continue to be encouraged.

We identified that 68 and 49 percent of the women's health apps reviewed did not have any star ratings or feedback comments. Evidence from this review and related articles clearly indicates that users are increasingly concerned about whether apps come from are reputable and legitimate sources. One review concluded that smartphone apps developed by experts are generally considered to be more preferable than those from unknown or less reputable sources [6].

It should also be considered that some training may be needed to help users get the most out of ST applications. While adolescents have a tendency to pick up the applications quickly, there is evidence that older adults may be less efficient and that ST applications should ideally be age-specific [42]. It is also clear that adherence in terms of daily use of the ST intervention should also be considered in future studies, as shown in the weight loss interventions described [22, 30]. Further work is also needed to determine “how long” users are likely to comply with the mobile health intervention; that is, what is the maximum number of weeks [6].

Finally, in terms of specific health outcomes, eight studies found that mobile medical applications helped to support weight loss [21, 23, 27–30, 32, 33]. However, it should be considered that the frequency of messages delivered may have had some effect on this [30]. Equally, the downloading of other health apps, alongside the one being tested may skew results [26]. While one published RCTs tested the effects of a nutritional (iodine) SMS intervention on markers of health (urinary iodine concentrations) dietary changes made were probably not substantial enough to generate an effect [22]. Consequently, future studies testing the effects of supplement compliance of fortified food consumption are warranted, in relation to app use and markers of nutritional well-being.

## 5. Conclusions

Overall the number of mobile medical applications developed specifically for aspects of women's health is rising but there is much yet to be done. Clearly more gender-specific, personalised apps are needed, for example, for mental health issues and heart disease. Given the small number of RCTs published for key areas of women's health, clearly further studies are warranted, particularly in relation to embedding and using apps in health care settings. Based on the feedback analysis, women are looking for apps that are easy to use, motivational, and, most importantly of all, trustworthy and evidence-based.

## Figures and Tables

**Table 1:** Medline and Web of Knowledge search terms.

(1) Women AND Weight AND Cellular phone OR mobile phone OR smartphone OR smart phone OR iPhone OR android phone OR blackberry OR Windows mobile OR mobile medical apps.	

(2) Women AND Diabetes AND Cellular phone OR mobile phone OR smartphone OR smart phone OR iPhone OR android phone OR blackberry OR Windows mobile OR mobile medical apps.	

(3) Women AND Heart Disease AND Cellular phone OR mobile phone OR smartphone OR smart phone OR iPhone OR android phone OR blackberry OR Windows mobile OR mobile medical apps.	

(4) Women AND Osteoporosis OR Bone Health AND Cellular phone OR mobile phone OR smartphone OR smart phone OR iPhone OR android phone OR blackberry OR Windows mobile OR mobile medical apps.	

(5) Women AND Breast Cancer AND Cellular phone OR mobile phone OR smartphone OR smart phone OR iPhone OR android phone OR blackberry OR Windows mobile OR mobile medical apps.	

(6) Women AND Nutrition OR Diet AND Cellular phone OR mobile phone OR smartphone OR smart phone OR iPhone OR android phone OR blackberry OR Windows mobile OR mobile medical apps.	

(7) Women AND Depression OR Mental Health AND Cellular phone OR mobile phone OR smartphone OR smart phone OR iPhone OR android phone OR blackberry OR Windows mobile OR mobile medical apps.	

(8) Women AND Pregnancy AND Cellular phone OR mobile phone OR smartphone OR smart phone OR iPhone OR android phone OR blackberry OR Windows mobile OR mobile medical apps.	

**Table 2:** Mobile medical applications and women's health.

Health outcome, publication, and location	Study population	Methods	Health application	Findings
Breast cancer				
Lakkis et al. [20] (2011), Lebanon	*n* = 385 females aged 40–75 years with a Health Insurance Plan	Prospective RCT. Randomized to two subgroups receiving SMS mobile phone texts: (1) to do a mammogram or (2) containing information about mammogram screening.	SMS tests either inviting women to do a mammogram or containing information about mammograms.	31% from group 1 and 32% from group 2 did a mammogram during the 6 months after intervention.

Heart health				
Park and Kim [21] (2012), Republic of Korea	*n* = 67 postmenopausal women	12-week RCT. Participants were asked to record their waist circumference and body weight, diet, and exercise levels using a weekly diary through the internet or by cellular phone.	Participants received weekly SMS/internet reminders about diet and exercise.	Systolic and diastolic blood pressure decreased by 6.5 and 4.6 mmHg in the intervention group. No significant changes were observed in the control group. Total cholesterol also reduced by 12.9 mg/dL in the intervention group but increased by 1.5 mg/dL in the control group.

Nutrition				
Mehran et al. [22] (2012), Iran	*n* = 205 females ≥ 18 yrs.	6-week RCT. Randomized to an intervention group that received text messages to enhance knowledge, attitudes, and practice concerning iron deficiency and iodized salt consumption.	The intervention group received daily text messages over 6 weeks. Urinary iodine concentration was measured at baseline and 8 weeks to see if there were any improvements.	Knowledge significantly improved by the end of the study (*P* = 0.004) but urinary iodine levels did not increased.
Norman et al. [23] (2013), USA	*n* = 52 overweight/obese adults, 80% female	4-month RCT. Randomized to intervention group receiving 2–5 weight management texts daily or a usual care comparison group.	Changes in fruit and vegetable intake and body weight were also measured.	Text messaging led to significant improvements in fruit and vegetable intake and eating behavior inventory scores.

Pregnancy				
Evans et al. [35] (2012), USA	*n* = 123, average age 27.6 years	RCT. Underserved pregnant women and new mothers were randomized to receive text messages to change their health, health beliefs, practices and behaviors to improve clinical outcomes, or to continue with usual health care.	Text4baby delivers text messages (https://text4baby.org/) to pregnant women and new mothers targeting underserved women facing health disparities.	There was an improvement of attitudes toward alcohol consumption from baseline to followup (*P* = 0.029).
Naughton et al. [24] (2012), UK	*n* = 207 pregnant mothers	11-week RCT. Women randomized to receive (1) a tailored self-help leaflet and 11-week tailored text messages or (2) a nontailored self-help leaflet.	11-weeks of tailored text messaging to quit smoking (MiQuit).	Those receiving the tailored text messages were more likely to set a quit date (*P* = 0.04) than controls.
Jareethum et al. [25] (2008), Thailand	*n* = 68 pregnant mothers	28-week RCT. Women randomized to receive prenatal support text messages sent from 28 weeks of pregnancy or a control group.	Two messages received per week from 28 weeks.	The confidence level was higher (*P* = 0.001) and anxiety level was lower (*P* = 0.002) amongst women receiving the text messages.

Weight loss				
Brindal et al. [26] (2013), Australia	*n* = 58 overweight and obese women	8-week RCT. Allocated to a weight loss or control group.	With the support of an app the intervention group received information about meal replacement programme.	The weight loss difference between groups was not significant (*P* = 0.08) although women in the intervention reported a greater increase in positive mood (*P* = 0.012)
Carter et al. [27] (2013), UK	*n* = 128 overweight volunteers, 33% females	6-month RCT. Randomized to a smartphone application or a website or paper diary weight loss intervention.	The app was used to self-monitor diet and activity and feedback was provided via weekly message.	Body mass index reductions after 6 months were the highest amongst the app users (−1.6 kg/m^2^), followed by the diary group (−1.0 kg/m^2^) and the website group (−0.5 kg/m^2^), indicating that this app was acceptable and feasible.
Donaldson et al. [28] (2013), UK	*n* = 23 overweight and obese females	12-week RCT. Randomized to receive tailored practitioner weight loss feedback or weight checks only (control).	Patients texted their weight loss progress to practitioners and received tailored feedback.	Body weight, BMI, and waist circumference all reduced significantly in the intervention compared with the control group.
Napolitano et al. [29] (2013), USA	*n* = 52 students (87% female)	8-week RCT. Randomized to (1) Facebook, (2) Facebook + text messaging, and personalised feedback (3) control group.	Messages were received over 8 weeks with weight loss measured at 4 and 8 weeks.	The Facebook + messaging group lost significantly more weight (−2.4 kg) after 8 weeks compared with the other branches.
Norman et al. [23] (2013), USA	*n* = 52 overweight/obese adults, 80% female	4-month RCT. Randomized to intervention group receiving 2–5 weight management texts daily or a usual care comparison group.	Changes in fruit and vegetable intake and body weight were also measured.	Text messaging led to significant improvements in body weight.
Park and Kim [21] (2012), Republic of Korea	*n* = 67 postmenopausal women	12-week RCT. Participants were asked to record their waist circumference and body weight, diet, and exercise levels using a weekly diary through the internet or by cellular phone.	Participants received weekly SMS/internet reminders about diet and exercise.	Waist circumference and body weight significantly decreased by 3.0 cm and 2.0 kg at 12 weeks compared with baseline. Increases were found in the control group.
Shapiro et al. [30] (2012), USA	*n* = 170 overweight and obese adults, 65% females	12-month RCT. Randomized to receive daily interactive and personally weight-relevant text messages or monthly e-newsletters.	Daily weight-relevant SMS and MMS received 4 times/day over 12 months.	Participants with greater adherence lost more weight 6 (*P* = 0.39) and 12 months (*P* = 0.23) than loss who were less adherent. Text messaging could be a useful adjunct to weight loss treatments.
Turner-McGrievy and Tate [31] (2011), USA	*n* = 96 overweight females	6-month RCT. Assigned to Podcast-only or Podcast + Mobile groups.	The Podcast + Mobile group uses a diet and physical activity monitoring app on the mobile devise and interacted with study counsellors using twitter.	Prompting and mobile communication via twitter did not enhance weight loss.
Haapala et al. [32] (2009), Finland	*n* = 125 overweight 22–44 year olds, 96 females	12-month RCT. Randomized to (1) use a mobile phone operated weight loss programme or (2) control	Participants received texts messages over 12 months and instructed on how to reduce food intake with daily weight reporting and tailored feedback.	After 12 months the experimental group had lost significantly more weight than the control (*P* = 0.006)
Patrick et al. [33] (2009), USA	*n* = 75 overweight men and women, 52% females	4-month RCT. Randomized to receive (1) printed materials about weight control, (3) MMS, and SMS messaging intervention.	Participants received personalised MMS and SMS messages were send 2–5 times per day.	The group receiving the messages lost more weight (−1.97 kg; *P* = 0.02) compared with the control group by the end of the study.

BMI: body mass index; MMS: multimedia messaging service; RCT: randomized controlled trial; SMS: short messaging service.

**Table 3:** Examples of commentaries from women's health apps.

Overall positive comments	Room for improvement
Easy to use. Great for keeping motivated and focused. Information was easy to digest. Information was very helpful. Makes my life easier.	Data could not be transferred into new versions. It did not tell me anything new. Graphs did not download. Information was out of date. Information was slow to download. More zoom would be useful. Needs extra graphics. Not certified/sure who developed it. Not compatible with iPad.
